# Cross-sectional association study of hedonic hunger, self-control, cognitive distortion, and well-being with adiposity measures among a sample of urban Malaysian adults

**DOI:** 10.1186/s40359-024-01680-2

**Published:** 2024-04-08

**Authors:** Yee-How Say, Mimi Shamirah Nordin, Alvin Lai Oon Ng

**Affiliations:** 1 Department of Biological Sciences, School of Medical and Life Sciences, Sunway University, Subang Jaya, Selangor Malaysia; 2 Department of Psychology, School of Medical and Life Sciences, Sunway University, Subang Jaya, Selangor Malaysia

**Keywords:** Adiposity, Obesity, Anthropometric measurement, Body composition, Psychometrics, Hedonic hunger

## Abstract

**Background:**

We assessed the association of hedonic hunger, self-control (impulsivity and restraint), cognitive distortion (CD), and well-being with adiposity measures such as waist circumference (WC), waist-to-hip ratio (WHR), waist-to-height ratio (WHtR), body mass index (BMI), total body fat (TBF), subcutaneous fat (SF), visceral fat level (VFL), skeletal muscle percentage (SM), and resting metabolism (RM), among a sample of urban Malaysian adults at Sunway University and Sunway College, Selangor, Malaysia.

**Methods:**

Among 186 participants (M/F = 51/135; aged 22.1 ± 5.0), psychometrics were assessed using Power of Food Scale (PFS), Brief Self-Control Scale, CD Questionnaire (CD-Quest), and WHO-5 Well-being Index. Blood pressures, anthropometrics and body compositions were also measured using standard methods and bioimpedance.

**Results:**

Men had significantly higher well-being, but lower overall self-control, impulsivity and Food Available hedonic hunger. Those with moderate/severe CD had higher odds ratio (OR) of having high central adiposity, compared with those with absent/slight CD (OR: 2.52;95% CI: 1.14, 5.61; *p* = 0.023 for WC and OR: 2.50; 95% CI: 1.19, 5.23; *p* = 0.015 for WHR). Higher CD and PFS scores were strongly significantly correlated with higher systolic blood pressure (SBP), WC, WHR, WHtR, BMI, TBF, SF, VFL and RM. Lower self-control was weakly correlated with higher WC, while lower impulsivity and restraint were weakly correlated with higher VFL. Those who were overweight, obese, and in high TBF class had significantly higher PFS Aggregate Factor scores. Food Available and Food Present scores, but not Food Tasted, were also significantly higher among overweight participants.

**Conclusions:**

Higher hedonic hunger and CD were associated with higher SBP and all adiposity measures. Overweight participants had higher hedonic hunger in the context of ready availability and physical presence of highly palatable foods. Lower self-control was weakly correlated with higher central adiposity; lower impulsivity and restraint were weakly correlated with higher visceral adiposity. These findings have provided some insights into the cognitive factors underlying adiposity.

## Abstract (plain language summary)

Higher recreational eating and irrational thinking, while lower self-control, may lead to higher fat accumulation and increased body weight among urban Malaysian adults.

## Introduction

Obesity, a global epidemic and public health crisis, is a multifactorial disorder caused by the interaction between genetics and the obesogenic environment [1]. The obesogenic environment - defined as “an environment that promotes high energy intake and sedentary behavior” that includes foods that are available, affordable, accessible and promoted [2] - highly influences the genetic susceptibility of obesity [1]. Appetite has been identified as the mediator of genetic susceptibility to the environment, a concept known as the behavioral susceptibility theory (BST) [3]. Excessive consumption of energy sources because of abnormal eating and appetitive behaviors might be one of the major risk factors for obesity. Food intake is regulated by two complementary drives: the homeostatic pathway, which motivation to eat follows the depletion of energy stores and thus controls the energy balance, and the hedonic pathway, which can override the former through increasing the desire for the consumption of highly palatable foods (such as high-fat and high-sugar diets) during the periods of relative energy abundance [4].

The obesogenic food environment may begin to influence psychological processes (e.g., thoughts, feelings, motivations) even when food intake is not imminent or underway. Given that there are individual differences in the degree to which the obesogenic food environment affects people’s thoughts, feelings and behavior, the Power of Food Scale (PFS) was developed in 2009 by Lowe et al., to measure individuals’ differences in hedonic hunger [5]. As the PFS does not contain any items that pertain to the actual consumption of food, the assessment of the appetitive aspects of eating by this measure is dependent on the psychological drive, rather than actual food overconsumption [5, 6].

General self-control, referring the ability to refrain from acting on undesired behavioral tendencies [7], is an important psychological factor that underlies the physiological and behavioral responses to the obesogenic environment, which include appetite and food intake [8]. Two psychological distinct domains of self-control - impulsivity (predisposition towards rapid, unplanned reactions to stimuli, without regard to the negative consequences [9]) and restraint (tendency to reflect and deliberate before acting [10])– are important in understanding weight-related behavior. Two meta-analyses conclude that dispositional (trait) self-control is only weakly related to eating behavior and weight control [11], while impulsivity is positively associated with BMI [12].

Dysfunctional cognitions are associated with obesity via unhealthy eating behaviors [13]. Cognitive distortion, one of the dysfunctional cognitive processes, are common thoughts that happen quickly, involuntarily, and in a distorted manner [14]. Some specific types of cognitive distortion are experienced by individuals with obesity, e.g. when they think that the desire to eat is irresistible (“magnification”), that they are “losers” because they are obese (“labeling”) or that people reject them because they are overweight (“mind reading”) [15]. Previous studies have found that individuals with obesity, especially those with comorbid binge eating disorder, experience more of some types of cognitive distortions than individuals of normal weight [16, 17].

The bidirectional association between obesity and depression has been documented in previous systematic reviews [18, 19], but remains controversial, as a meta-analysis found small effect sizes [20]. Identifying depression may improve weight loss in patients with obesity, and weight loss may improve the symptoms of depression, suggesting that rapid screening for both diseases is essential to health promotion and prevention of further disease [21]. As such, the WHO-5 Wellbeing can be used, as although it is a generic wellbeing scale without any diagnostic specificity, it has been shown to be useful index to screen for possible association of psychological distress with adiposity measures [21].

Gender is a recognized difference in the field of psychology, in terms of emotional, motivational, or cognitive differences [22]. There have been reported studies on the gender differences in the four psychometric measures in this study, namely hedonic hunger, self-control, cognitive distortion and well-being. Women were more prone to hedonic hunger [23–26] and had generally lower WHO-5 well-being [27], but had higher self-control [28] and experienced lesser cognitive distortion related to externalizing problems [29, 30] compared with men/boys.

To the best of our knowledge, there is no study investigating the association of psychometric measures, i.e. hedonic hunger, self-control, cognitive distortion and well-being with adiposity measures among the Malaysian population. In addition, motivations to consume food differ from one country to another, due to the differences in ethnic, and cultural and social backgrounds. Given that the psychometric measures have been previously associated with adiposity measures as mentioned above, therefore, the main aim of this study was to investigate the association of the four psychometric measures with anthropometric and body composition measures, such as waist circumference (WC), waist-to-hip ratio (WHR), waist-to-height ratio (WHtR), visceral fat level (VFL), and body mass index (BMI). The participants were a sample of Malaysian adults at Sunway University and Sunway College in Sunway City (3.0684° N, 101.6025° E), an urbanized environment where foods are highly available, affordable, accessible and promoted here.

## Materials and methods

### Participant recruitment and ethical approval

Participants in this cross-sectional study were recruited from the students and staff of Sunway University and Sunway College, Sunway City, Selangor, Malaysia, from June– December 2022 (without COVID-19 movement restrictions) by convenience sampling, through publicity materials around campus and word-of-mouth. Questionnaire completion by the participants and physical measurements were conducted on the spot at Sunway University. Participants must meet the following inclusion criteria: (1) Malaysian, aged 18–80 years; (2) no current major medical condition (e.g., cancer, liver or kidney disease); (3) no history of or current endocrine pathology (Cushing syndrome, pseudohypoparathyroidism, etc.); (4) no history of neurological disorder or injury (e.g. stroke, or seizures; loss of consciousness > 10 min); (5) no history of or current serious psychological disorder (i.e., severe depression or anxiety, substance use disorder, psychoses, bipolar disorder); (6) not currently pregnant or breastfeeding; (7) no impaired sensory function (e.g., visually impaired); (8) no physical activity contraindication; (9) not taking any medication that impacts weight and appetite (e.g., mirtazapine, prednisone); (10) no history of syndromic obesity (Prader Willi, Alström, Laurence-Moon Biedle syndrome, etc.). Screening of the inclusion criteria was performed during the participant’s first visit and if eligible, participants were assigned a subject ID. Briefing on how to answer the online questionnaires was performed, clinical, and anthropometric measurements were taken, and participants were given a reimbursement upon completion.

Using the Raosoft sample size online calculator (http://www.raosoft.com/samplesize.html), a minimum sample size of 187 is needed to achieve a 6% margin of error, 90% confidence level, Sunway University and Sunway College population size of 22,000, and a 50% response distribution.

Ethical approval was obtained from the Sunway University Research Ethics Committee (SUREC 2022/008), all participants signed informed consent forms, and the study was conducted in accordance with the Declaration of Helsinki.

### Sociodemographic and lifestyle factors questionnaire

Sociodemographics, i.e. self-identified Malaysian ethnicity (Malay/Chinese/Indian), age, highest education level (primary/secondary/tertiary), marital status (single/married/divorced or widowed) and monthly household income (B40/M40/T20) were assessed. According to the Department of Statistics Malaysia (2019), monthly household income is defined as total gross income before taxes, received by all members of a household [for students, unemployed or financially-dependent individuals: parents’ household income; for employed and financially-independent individuals: the combined (own, spouse’s, children’s household income)] [31]. The B40, M40 and T20 categories were ≤ MYR4,850, 4851-10,960, and ≥ 10,961 (approximately ≤ USD1065, 1066–2,406, and ≥ 2407), respectively [31].

### Psychometrics questionnaires

The Power of Food Scale (PFS; [5]) was used to assess the psychological impact of living in food-abundant environments. It measures appetite for, rather than consumption of, palatable foods, at three levels of food proximity (food available, food present, and food tasted). PFS is a 15-item questionnaire presented on a five-point Likert scale ranging from 1 (do not agree at all) to 5 (strongly agree). All items are scored so that a higher item score indicated a greater responsiveness to the food environment. Scoring for Food Available, Food Present, Food Tasted scores were according to the rubrics outlined by Lowe et al., 2009 [5]. A Power of Food Aggregate Factor was also generated by averaging the scores of all 15 questions.

Self-regulation was assessed using the Brief Self-Control Scale (BSCS; [7]), a 13-item self-report questionnaire that assesses trait levels of self-control, with a focus on a person’s ability to override an inner urge and refrain from acting on it. It is rated from 1 (“not at all like me”) to 5 (“very much like me”) and has high internal consistency, test-retest reliability, and construct validity. The original BSCS measures a single self-control construct [7], which represents the tendency to be disciplined and abrogate impulses. A summation of BSCS total scores (Q2, 3, 4, 5, 7, 9, 10, 12, 13 were reverse-scored) was generated (BSCS Total Score). As Maloney et al. [32] found that the BSC measures impulsivity and restraint as two distinct factors, BSCS Impulsivity and Restraint Scores were generated from summation of scores for Q5R, 9R, 10R, 12R, 13R, and Q1, 2R, 7R, 8, respectively. Impulsivity is related to acting on spontaneous thoughts and feelings, while restraint is related to self-discipline and resisting temptation.

Cognitive distortion (CD) is defined as an exaggerated or irrational thought pattern involved in the onset or perpetuation of psychopathological states, such as depression and anxiety [33]. The frequency and intensity of cognitive distortions was assessed using the 15-item CD Questionnaire (CD-Quest) [34]. Participants were instructed to indicate which cognitive distortions they had experienced in the past week, “how much [they] believed it in the exact moment it occurred” (i.e., intensity), and “how often it occurred during this past week” (i.e., frequency). Scoring rubrics were performed according to [34]. A summation of the CD-Quest scores was generated. The 1-25th, 26-50th, 51st– 75th, and 76th– 100th percentiles of CD-Quest scores were categorized as absent/minimal, slight, moderate, and severe magnitudes of CD, respectively [34]. A higher score indicates greater CD.

The five-item World Health Organization Five Well-Being Index (WHO-5) [35] was used as a measure of general wellbeing which asks respondents to rate their interest, engagement and mood. The raw score ranges from 0 to 25, with 0 representing the worst possible and 25 representing the best possible quality of life. A score below 13 indicates poor well-being.

### Clinical, anthropometric and body composition measurements

All measurements were conducted in the morning, before lunch time. Clinical measurements indicative of vascular health namely systolic blood pressure (SBP), diastolic blood pressure (DBP) and pulse rate were taken using an automated blood pressure monitor (HEM-7121, Omron, Japan) after the subjects had rested for 5 min. Height was measured using a wall-mounted stadiometer. Waist and hip circumferences were measured using a stretch-resistant tape that provided a constant 100 g tension, at the midpoint between the lower margin of the least palpable rib and the top of the iliac crest and around the widest portion of the buttocks, respectively [36]. The waist-hip ratio (WHR) and waist-to-height ratio (WHtR) were calculated by dividing waist circumference by hip circumference and height, respectively. A bioimpedance body composition scale (Omron HBF-375) was used to determine weight, body mass index (BMI; kg/m^2^), total body fat (TBF; %), visceral fat level (VFL; %), subcutaneous fat (SF; %), skeletal muscle percentage (SM; %) and resting metabolism rate (RM; kcal). Projected body age was also generated based on the proprietary formula that takes RM, weight and TBF into consideration [37]. It provides a guide to judge whether the body age is above or below the average of a participant’s actual age. The cutoff points for overweight, obesity, high TBF, high VFL, high SM, high WC, high WHR and high WHtR are ≥ 23 kg/m^2^ [38]; ≥27.5 kg/m^2^ [38]; 20% (men) or 30% (women) [37]; 10% [37]; 35.8% (men) or 28% (women) [37]; 90 cm (men) or 80 cm (women) [38]; 0.90 (men) or 0.85 (women) [36]; and 0.50 [39], respectively.

### Statistical analysis

Statistical analysis of the data was performed using IBM SPSS Statistics for Windows 26.0 (IBM Corp., Armonk, NY, USA). Descriptive statistics for the categorical variables (demographic characteristics) were presented in terms of frequency and percentage. The conformity of the numerical variables to normal distribution was determined by the Kolmogorov-Smirnov test, where *p* > 0.05 indicates normally-distributed data. The Mann–Whitney *U* test (*U*) was used in the comparison of two independent groups that did not have a normal distribution, while the Kruskal–Wallis test was used in the comparison of more than two groups. Examination of the relationships between the scales was determined by partial rank correlation test, by first performing a Spearman bivariate correlation for all variables and adding the Spearman rank correlation coefficients into a new file, and then performing partial correlation on the desired variables by using the newly created Spearman correlation coefficients, controlling for confounding socio-demographic factors, i.e. age, gender, ethnicity, monthly household income, education level and marital status. In the interpretation of the correlation coefficient, it was determined as a “very weak correlation, if < 0.2”, a “weak correlation between 0.2 and 0.4”, a “moderate correlation between 0.4 and 0.6”, a “high correlation between 0.6 and 0.8”, and “0.8 > very high correlation”. Binary logistic regression analysis was used to test the association of well-being and CD with anthropometric and body composition classes, unadjusted or adjusted for confounding socio-demographic factors, i.e. age, gender, ethnicity, monthly household income, education level and marital status. Nonnormally distributed data were transformed by log or reciprocal transformation before analysis of covariance (ANCOVA). ANCOVA was performed between anthropometric and body composition groups using univariate General Linear Model, adjusted for confounding socio-demographic factors, i.e. age, gender, ethnicity, monthly household income, education level and marital status. The *p*-value of < 0.05 was considered as statistically significant.

## Results

Out of 300 participants recruited for the study, 186 participants have completed the questionnaires in entirety and had all measurements recorded (dropout rate: 38%). The mean age of the overall participants was 22.09 ± 5.04 years (men: 22.12 ± 4.31; women: 22.07 ± 5.30), with age range: 18–49 years and men: women ratio 1: 2.65. Table 1 shows that the majority of them were of Chinese ethnicity, students between 18 and 24 ages, currently pursuing a tertiary education level, single, and were from the M40 monthly household income category. The frequency distribution of sociodemographics (ethnicity, age, education level, marital status, monthly household income), TBF, SM, WC classes and CD-Quest categories did not differ significantly between genders (Table 1). However, there were significantly more men who were in the prehypertension or hypertension class I, overweight, obese, high VFL, WHR and WHtR, and good well-being categories (Table 1).

**Table 1:** Socio-demographic characteristics, blood pressure, anthropometric and body composition classifications according to gender

	Men (*n* = 51)		Women (*n* = 135)		Chi-square test				
	n	%	n	%	*x* ^2^	*p*	df	CC^§^	phi
*Ethnicity*									
Malay	6	11.8	20	14.8	0.304	0.859	2	-	0.04
Chinese	41	80.4	104	77.0					
Indian	4	7.8	11	8.1					
*Age groups*									
18–24	42	82.4	122	90.4	3.043	0.218	2	-	0.128
25–30	5	9.8	5	3.7					
> 30	4	7.8	8	5.9					
*Education level*									
Secondary	6	11.8	14	10.4	0.075	0.784	1	0.993	0.02
Tertiary	45	88.2	121	89.6					
*Marital status*									
Single	48	94.1	129	95.6	0.166	0.684	1	0.980	− 0.03
Married	3	5.9	6	4.4					
*Monthly household income*									
B40	17	33.3	33	24.4	4.751	0.093	2	-	0.16
M40	20	39.2	77	57.0					
T20	14	27.5	25	18.5					
*Hypertension class*									
Normal	18	35.2	117	86.7	52.558	< 0.001**	2	-	0.532
Pre-hypertension	28	54.9	18	13.3					
Stage I hypertension	5	9.8	0	0					
*BMI Classification (Overweight)*									
Non-overweight	31	60.8	102	75.6	3.964	0.046*	1	0.07	− 0.146
Overweight	20	39.2	33	24.4					
*BMI Classification (Obese)*									
Non-obese	43	84.3	130	96.3	8.176	0.004**	1	0.011*	− 0.21
Obese	8	15.7	5	3.7					
*TBF Classification*									
Normal	32	62.7	95	70.4	0.994	0.319	1	0.412	− 0.073
High	19	37.3	40	29.6					
*VFL Classification*									
Normal	41	80.4	131	97.0	14.734	< 0.001**	1	< 0.001**	− 0.281
High	10	19.6	4	3.0					
*SM Classification*									
Normal	30	58.8	88	65.2	0.646	0.422	1	0.527	− 0.059
High	21	41.2	47	34.8					
*WC Classification*									
Normal	38	74.5	108	80.0	0.661	0.416	1	0.54	-0.06
High	13	25.5	27	20.0					
*WHR Classification*									
Normal	30	58.8	105	77.8	6.682	0.010*	1	0.016*	− 0.19
High	21	41.2	30	22.2					
*WHtR Classification*									
Normal	33	64.7	107	79.3	4.212	0.040*	1	0.063	− 0.15
High	18	35.3	28	20.7					
*WHO-5 Well-being Category*									
Good	44	86.3	90	72.0	7.066	0.008**	1	0.013	0.195
Poor	7	13.7	45	28.0					
*CD-Quest Category*									
Absent/Slightly	24	47.1	71	52.6	0.454	0.501	1	0.611	− 0.049
Moderately/Severely	27	52.9	64	47.4					

BMI: Body Mass Index; TBF: Total Body Fat; VFL: Visceral Fat Level; SM: Skeletal Muscle Percentage; WC: Waist Circumference; WHR: Waist-Hip Ratio; WHtR: Waist-Height Ratio

^**§**^Continuity correction *p*-value; only computed for 2 × 2 table

*p-value is significant at the 0.05 level (2-tailed)

**p-value is significant at the 0.01 level (2-tailed)

Cronbach’s alpha values were 0.853, 0.963, 0.794, 0.855, 0.793, 0.784, and 0.908 for WHO well-being, CDQuest, BSCS Total, Food Available, Food Present, Food Tasted, and Power of Food Aggregated Factor, respectively, indicating a high level of internal consistency for all scale and subscales with this specific sample.

Table 2 shows that the WHO well-being, BSCS Total, BSCS Restraint and Food Available scores were significantly different between genders (albeit with small effect sizes), where men had significantly higher well-being, but lower overall self-control, impulsivity and hedonic hunger toward food availability.

**Table 2:** Psychometric measures of overall participants and between genders

Psychometrics	Men (*n* = 51)	Women (*n* = 135)	Total (*N* = 186)	U/t	*p*	df^¥^	Effect size^§^
WHO-5 Well-being Index Score	17 (6, 25)	15 (1, 25)	15 (1, 25)	2443.50	0.002**	-	− 0.224
CD-Quest Score	15 (1, 54)	13 (0, 56)	14 (0, 56)	3140.50	0.356	-	− 0.068
BSCS Total Score^¥^	36.12 ± 7.633	38.69 ± 7.670	37.98 ± 7.725	-2.042	0.043*	184	− 0.336
BSCS Impulsivity Score	14 (6, 21)	16 (6, 25)	15 (6, 25)	2656.00	0.016*	-	− 0.177
BSCS Restraint Score	11 (6, 19)	10 (4, 18)	10 (4, 19)	3120.50	0.322	-	− 0.073
Food Available	2.33 (1, 5)	3.00 (1, 5)	2.67 (1, 5)	2743.00	0.032*	-	− 0.157
Food Present	3.00 (1, 5)	3.25 (1, 5)	3.25 (1, 5)	3309.00	0.683	-	− 0.030
Food Tasted	2.80 (1, 5)	3.20 (1, 5)	3.10 (1, 5)	3110.50	0.310	-	− 0.081
PFS Aggregate Factor^¥^	2.77 ± 0.855	2.94 ± 0.823	2.90 ± 0.833	-1.289	0.199	184	− 0.204

All values are median (minimum, maximum) unless otherwise stated; *U* and *p*-values are by Mann-Whitney *U* test

^¥^Values are mean ± SD, *t* and *p*-values by independent *t*-test

*p-value is significant at the 0.05 level (2-tailed)

^¥^df computed only for independent *t*-test

^§^Effect size for Mann-Whitney *U* test is derived from Z/√N and is Cohen’s D value for independent *t*-test

Table 3 shows that those with moderate/severe CD had an around two-fold significantly higher risk of having a high WHR [OR (95% CI): 1.937 (1.005, 3.736)]; this significance remained true after controlling for sociodemographics [OR (95% CI) = 2.498 (1.193, 5.228). Similarly, after controlling for sociodemographics, those with moderate/severe CD also had a 2.5-fold significantly higher risk of having high WC [OR (95% CI) = 2.524 (1.135, 5.614)]. Well-being was not associated with anthropometric and body composition classes (Table 3).

**Table 3:** Association between well-being and cognitive distortion with anthropometric and body composition classes

Anthropometric/Body Composition Classes	WHO-5 Well-being Index Category		CD-Quest Category	
	Unadjusted	Adjusted^§^	Unadjusted	Adjusted^§^
*BMI Classification (Overweight)*				
Non-overweight	1.00	1.00	1.00	1.00
Overweight	0.680 [0.324, 1.430]	0.795 [0.361, 1.748]	1.120 [0.592, 2.117]	1.149 [0.589, 2.243]
*p*	0.310	0.568	0.728	0.684
*BMI Classification (Obese)*				
Non-obese	1.00	1.00	1.00	1.00
Obese	0	0	0.887 [0.287, 2.748]	0.884 [0.268, 2.916]
*p*	0.999	0.999	0.836	0.839
*TBF Classification*				
Normal	1.00	1.00	1.00	1.00
High	1.064 [0.536, 2.110]	1.151 [0.550, 2.30]	0.918 [0.495, 1.703]	1.009 [0.527, 1.934
*p*	0.859	0.709	0.785	0.977
*VFL Classification*				
Normal	1.00	1.00	1.00	1.00
High	0.183 [0.023, 1.432]	0.293 [0.034, 2.507]	1.048 [0.352, 3.114]	1.136 [0.352, 3.670]
*p*	0.106	0.263	0.933	0.831
*SM Classification*				
Normal	1.00	1.00	1.00	1.00
High	0.999 [0.514, 1.942]	0.976 [0.472, 2.016]	0.975 [0.537, 1.772]	0.818 [0.432, 1.547]
*p*	0.997	0.947	0.935	0.536
*WC Classification*				
Normal	1.00	1.00	1.00	1.00
High	1.530 [0.725, 3.232]	1.653 [0.724, 3.772]	2.020 [0.985, 4.143]	2.524 [1.135, 5.614]
*p*	0.265	0.233	0.055	0.023*
*WHR Classification*				
Normal	1.00	1.00	1.00	1.00
High	1.104 [0.542, 2.247]	1.419 [0.643, 3.132]	1.937 [1.005, 3.736]	2.498 [1.193, 5.228]
*p*	0.786	0.386	0.048*	0.015*
*WHtR Classification*				
Normal	1.00	1.00	1.00	1.00
High	1.020 [0.486, 2.139]	1.224 [0.544, 2.756]	1.189 [0.610, 2.315]	1.316 [0.641, 2.703]
*p*	0.958	0.625	0.612	0.454

Values are Odds Ratio [Confidence Interval]; by binary logistic regression analysis

^**§**^Adjusted for: age, gender, ethnicity, monthly household income, education level and marital status

*p-value is significant at the 0.05 level (2-tailed)

**p-value is significant at the 0.01 level (2-tailed)

To further assess the relationship between psychometrics and blood pressures, anthropometric and body composition measurements, partial rank correlation test was performed, controlling for sociodemographics (Table 4). Poorer well-being was significantly correlated with higher TBF and lower SM, but not others. Higher CD and PFS scores were strongly significantly correlated with higher SBP, anthropometrics, body compositions, RM and projected body age, except for SM (all *r* values > 0.4, *p* < 0.001; Table 4). Lower self-control was marginally correlated with higher WC (*r* = -0.386, *p* = 0.047), while lower impulsivity and restraint were marginally correlated with higher VFL (*r* = -0.397, *p* = 0.04; *r* = -0.386, *p* = 0.047, respectively; Table 4).

**Table 4:** Correlation between psychometrics (well-being, cognitive distortion, self-control and hedonic hunger) with blood pressures, anthropometrics and body compositions

Psychometrics		SBP	DBP	WC	WHR	Weight	WHtR	BMI	TBF	SF	VFL	SM	RM	Body age
WHOWell-beingScore	*r*	0.369	0.178	− 0.074	0.042	− 0.097	− 0.102	− 0.061	− 0.448	− 0.377	− 0.079	0.513	0.069	− 0.21
	*p*	0.058	0.374	0.714	0.837	0.632	0.613	0.763	0.019*	0.053	0.695	0.006**	0.731	0.294
CD-Quest Score	*r*	0.618	0.667	0.748	0.813	0.656	0.676	0.62	0.526	0.515	0.643	0.416	0.707	0.56
	*p*	0.001**	< 0.001**	< 0.001**	< 0.001**	< 0.001**	< 0.001**	0.001**	0.005**	0.006**	< 0.001**	0.031*	< 0.001**	0.002**
BSCS Total Score	*r*	− 0.056	− 0.099	− 0.386	− 0.362	− 0.277	− 0.373	− 0.345	− 0.133	− 0.213	− 0.37	− 0.024	− 0.27	− 0.301
	*p*	0.781	0.625	0.047*	0.064	0.162	0.055	0.078	0.508	0.287	0.058	0.905	0.173	0.128
BSCSImpulsivity	*r*	− 0.153	− 0.21	− 0.378	− 0.346	− 0.292	− 0.356	− 0.366	− 0.001	− 0.058	− 0.397	− 0.147	− 0.262	− 0.29
	*p*	0.447	0.292	0.052	0.077	0.139	0.068	0.061	0.995	0.775	0.04*	0.465	0.186	0.142
BSCS Restraint	*r*	0.288	0.186	− 0.304	− 0.049	− 0.319	− 0.365	− 0.371	− 0.298	− 0.341	− 0.386	0.208	− 0.052	− 0.349
	*p*	0.144	0.353	0.123	0.81	0.105	0.061	0.057	0.131	0.081	0.047*	0.299	0.797	0.074
Food Available	*r*	0.409	0.332	0.822	0.696	0.81	0.827	0.857	0.655	0.706	0.834	0.211	0.772	0.848
	*p*	0.034*	0.091	< 0.001**	< 0.001**	< 0.001**	< 0.001**	< 0.001**	< 0.001**	< 0.001**	< 0.001**	0.29	< 0.001**	< 0.001**
Food Present	*r*	0.453	0.412	0.866	0.78	0.799	0.856	0.865	0.507	0.561	0.868	0.38	0.771	0.76
	*p*	0.018*	0.033*	< 0.001**	< 0.001**	< 0.001**	< 0.001**	< 0.001**	0.007**	0.002**	< 0.001**	0.051	< 0.001**	< 0.001**
Food Tasted	*r*	0.443	0.313	0.727	0.717	0.623	0.691	0.696	0.511	0.552	0.718	0.227	0.7	0.653
	*p*	0.021*	0.111	< 0.001**	< 0.001**	0.001**	< 0.001**	< 0.001**	0.006**	0.003**	< 0.001**	0.256	< 0.001**	< 0.001**
PFS Aggregate Factor	*r*	0.443	0.366	0.828	0.721	0.787	0.817	0.852	0.621	0.671	0.844	0.258	0.761	0.812
	*p*	0.021*	0.061	< 0.001**	< 0.001**	< 0.001**	< 0.001**	< 0.001**	0.001**	< 0.001**	< 0.001**	0.193	< 0.001**	< 0.001**

CD-Quest: Cognitive distortion questionnaire; SBP: Systolic blood pressure; DBP: Diastolic blood pressure; WC: Waist Circumference; WHR: Waist-Hip Ratio; WHtR: Waist-Height Ratio; BMI: Body Mass Index; TBF: Total Body Fat; SF: Subcutaneous Fat; VFL: Visceral Fat Level; SM: Skeletal Muscle Percentage; RM: Resting Metabolism

*r* and *p*-values by partial rank correlation, controlling for variables: age, gender, ethnicity, monthly household income, education level and marital status

*Correlation is significant at the 0.05 level (2-tailed)

**Correlation is significant at the 0.01 level (2-tailed)

When comparing means, those who belonged to the overweight, obese, and high TBF class had significantly higher PFS Aggregate Factor scores, while those with high SM had a significantly lower score (Table 5). Food Available score was also significantly higher among overweight and obese participants, while Food Present score was significantly higher among overweight and high TBF participants (Table 5). Albeit, the effect sizes of these significant scores were small (all η^2^ values less than 0.04). BSCS Total, Impulsivity and Restraint scores were not significantly different between all anthropometric and body composition classes (data not shown).

**Table 5:** ANCOVA analysis of Power of Food Scores between anthropometric and body composition classes

Anthropometric/Body Composition Classes	PFS			
	Food Available	Food Present	Food Tasted	Aggregated Score
*BMI Classification (Overweight)*				
Non-overweight	2.547 ± 0.079	3.000 ± 0.083	3.049 ± 0.081	2.815 ± 0.07
Overweight	2.850 ± 0.127	3.376 ± 0.134	3.167 ± 0.130	3.100 ± 0.116
*F*; *p*	4.013; 0.047*	5.577; 0.019*	0.586; 0.445	4.265; 0.040*
df; η^2^	1; 0.022	1; 0.030	1; 0.003	1; 0.023
*BMI Classification (Obese)*				
Non-obese	2.584 ± 0.068	3.071 ± 0.073	3.063 ± 0.070	2.855 ± 0.063
Obese	3.328 ± 0.268	3.582 ± 0.277	3.348 ± 0.266	3.440 ± 0.237
*F*; *p*	7.190; 0.008**	3.153; 0.078	1.064; 0.304	5.619; 0.019*
df; η^2^	1; 0.039	1; 0.017	1; 0.006	1; 0.031
*TBF Classification*				
Normal	2.542 ± 0.081	2.980 ± 0.085	3.023 ± 0.083	2.797 ± 0.074
High	2.829 ± 0.121	3.381 ± 0.126	3.211 ± 0.123	3.110 ± 0.109
*F*; *p*	3.777; 0.054	6.750; 0.010*	1.572; 0.212	5.491; 0.020*
df; η^2^	1; 0.021	1; 0.037	1; 0.009	1; 0.030
*VFL Classification*				
Normal	2.598 ± 0.069	3.081 ± 0.074	3.082 ± 0.071	2.870 ± 0.064
High	3.075 ± 0.265	3.425 ± 0.273	3.094 ± 0.262	3.220 ± 0.235
*F*; *p*	2.980; 0.086	1.458; 0.229	0.002; 0.963	2.040; 0.155
df; η^2^	1; 0.017	1; 0.008	1; 0.000	1; 0.011
*SM Classification*				
Normal	2.757 ± 0.084	3.220 ± 0.090	3.145 ± 0.086	3.004 ± 0.077
High	2.417 ± 0.112	2.910 ± 0.119	2.974 ± 0.115	2.709 ± 0.103
*F*; *p*	5.650; 0.019*	4.149; 0.043*	1.361; 0.245	5.107; 0.025*
df; η^2^	1; 0.031	1; 0.023	1; 0.008	1; 0.028
*WC Classification*				
Normal	2.619 ± 0.076	3.055 ± 0.081	3.074 ± 0.077	2.868 ± 0.070
High	2.681 ± 0.152	3.296 ± 0.158	3.114 ± 0.152	2.998 ± 0.137
*F*; *p*	0.130; 0.719	1.68; 0.183	0.054; 0.817	0.694; 0.406
df; η^2^	1; 0.001	1; 0.010	1; 0.000	1; 0.004
*WHR Classification*				
Normal	2.657 ± 0.080	3.074 ± 0.085	3.100 ± 0.081	2.900 ± 0.073
High	2.565 ± 0.135	3.192 ± 0.142	3.037 ± 0.136	2.885 ± 0.123
*F*; *p*	0.326; 0.569	0.486; 0.486	0.154; 0.695	0.012; 0.914
df; η^2^	1; 0.002	1; 0.003	1; 0.001	1; 0.000
*WHtR Classification*				
Normal	2.603 ± 0.078	3.058 ± 0.083	3.058 ± 0.079	2.859 ± 0.071
High	2.722 ± 0.141	3.256 ± 0.148	3.157 ± 0.141	3.010 ± 0.127
*F*; *p*	0.529; 0.469	1.327; 0.251	0.362; 0.548	1.029; 0.312
df; η^2^	1; 0.003	1; 0.007	1; 0.002	1; 0.006

All parameters were either transformed by log or reciprocal transformation to conform to the normal distribution of data in univariate General Linear Model analysis

Values are estimated marginal means ± standard error, generated after controlling for sociodemographic factor covariates: age, gender, ethnicity, monthly household income, education level and marital status

*p-value is significant at the 0.05 level (2-tailed)

**p-value is significant at the 0.01 level (2-tailed)

## Discussion

Men had significantly higher well-being, but lower overall self-control, impulsivity and hedonic hunger toward food availability. Gender differences in the aforementioned psychometrics were rather mixed in previous studies. Women had generally lower WHO-5 well-being than men among the general population in 26 European countries [27], and among health professionals in four European countries [40]. Among college students, self-control scores were indifferent between genders among Chinese college students [41, 42]. Women were more prone to hedonic hunger in previous studies [23–25, 43], but another study found no gender differences in the three domains and aggregate scores of PFS [26].

With regard to the significant association between CD and central adiposity/obesity as assessed by WHR and WC, in contrast, another study found that the CD-Quest Scores were not significantly different between morbidly-obese (BMI ≥ 40 kg/m^2^) and normal weight individuals [44]. When other eating disorder-specific CD questionnaires were used, one small study found that obese individuals, regardless of eating disorders, were found to have higher CD than non-obese individuals [16]. Meanwhile, two other small studies reported that obese individuals were less vulnerable to thought-shape fusion (imagination of the consumption of high-energy food generates the feeling of being fat and negative moral judgment) than normal weight individuals [45, 46].Thus, a concrete conclusion could not be made on the relationship between CD and adiposity/obesity, given the limited number of studies and small sample sizes. Nevertheless, these studies suggest that obese individuals, particularly those with binge eating disorder comorbidity, experience more of some types of cognitive distortions than normal weight individuals.

There is still no consensus yet on the association between PFS and current and future change in BMI. Small to moderate and statistically significant associations between PFS and current BMI have been identified [47, 48]. PFS domain-wise, overweight was associated with Food Available and Food Present among US young adults, but not Food Tasted and PFS Aggregate Score [49]. However, in a review of ten studies, Espel-Huynh et al. [50] concluded that there is little evidence for a relationship between the PFS and BMI, as majority of the studies found non-significant results between BMI and hedonic hunger in both men and women, with *r*-values ranging from 0.02 to 0.35. Nevertheless, they also concluded that PFS scores appear to decline over time among overweight/obese patients who underwent weight loss [50]. In the present study, moderate to very high correlations were observed between all three domains of PFS, PFS Aggregate Score and not only BMI, but also central, overall, visceral and subcutaneous adiposity. Means of Food Available, Food Present, PFS Aggregate Factor, but not Food Tasted, were also significantly higher among overweight participants. One of the possible reasons for the discrepancies in the results findings, as given by Espel-Huynh et al. [50], is that there are other psychological factors that work in tandem besides hedonic hunger to predict food intake beyond one’s caloric needs and subsequent increased adiposity. Indeed, in this study, we found that higher CD was strongly correlated with higher adiposity, lower self-control was weakly correlated with higher central adiposity, while lower impulsivity and restraint were weakly correlated with higher visceral adiposity. These indicate that dysfunctional cognitive processes may lead to loss of control over eating (dysfunctional eating behavior), which in turn will lead to increased adiposity or obesity [15].

Due to the several limitations in this study, the findings should be interpreted with caution. The one-time cross-sectional nature of the study does not allow for a cause–effect conclusion to be made. With a relatively small sample size and imbalanced age, gender and overweight/obese proportions, the study consisted of generally healthy, well-educated and almost ethnically-homogenous participants from an urban geographical area. Thus, our results may not necessarily extrapolate to the general multiethnic Malaysian adult population. Nevertheless, our study assessed a diverse range of psychometric measures related to adiposity, i.e. well-being, CD, self-control, hedonic hunger, and we also measured adiposity and cardiovascular measures comprehensively with 15 parameters of blood pressures, anthropometric, and body composition measurements. Future longitudinal studies should include how hedonic hunger and other psychometrics may change in individuals in the general population who are currently engaging in volitional efforts to reduce weight, either through dieting, exercise, or both.

## Conclusions

In conclusion, higher hedonic hunger and CD were significantly associated with higher blood pressures, anthropometrics and body compositions, particularly central adiposity. Hedonic hunger in the context of readily available and physical presence of highly palatable foods, were predictors of overall adiposity. Lower self-control was weakly correlated with higher central adiposity, while lower impulsivity and restraint were weakly correlated with higher visceral adiposity. These findings provide some insights into cognitive factors underlying overweight and obesity. Further evidence would be needed to support the connections between the psychological variables in this study and the anthropometry/body composition measures.

## Data Availability

Data analysed or generated in our study would be made available on contacting corresponding author.

## Abbreviations

BMI: Body Mass Index
BSCS: Brief Self-Control Scale
CD: Cognitive distortion
CD-Quest: Cognitive Distortion Questionnaire
CI: Confidence Interval
DBP: Diastolic blood pressure
OR: Odds Ratio
PFS: Power of Food Scale
RM: Resting Metabolism
SBP: Systolic Blood Pressure
SM: Skeletal Muscle Percentage
TBF: Total Body Fat
VFL: Visceral Fat Level
WC: Waist Circumference
WHR: Waist-Hip Ratio
WHtR: Waist-Height Ratio
